# *Morus alba* L. (Sangzhi) Alkaloids Promote Insulin Secretion, Restore Diabetic β-Cell Function by Preventing Dedifferentiation and Apoptosis

**DOI:** 10.3389/fphar.2022.841981

**Published:** 2022-03-03

**Authors:** Lei Lei, Yi Huan, Quan Liu, Caina Li, Hui Cao, Wenming Ji, Xuefeng Gao, Yaxin Fu, Pingping Li, Ruiping Zhang, Zeper Abliz, Yuling Liu, Shuainan Liu, Zhufang Shen

**Affiliations:** ^1^ Institute of Materia Medica, Chinese Academy of Medical Sciences and Peking Union Medical College, Beijing, China; ^2^ State Key Laboratory of Bioactive Substances and Functions of Natural Medicines, Key Laboratory of Polymorphic Drugs of Beijing, Institute of Materia Medica, Chinese Academy of Medical Sciences and Peking Union Medical College, Beijing, China; ^3^ Diabetes Research Center of Chinese Academy of Medical Sciences and Peking Union Medical College, Beijing, China; ^4^ CAMS Key Laboratory of Molecular Mechanism and Target Discovery of Metabolic Disorder and Tumorigenesis, Chinese Academy of Medical Sciences and Peking Union Medical College, Beijing, China; ^5^ Drug Delivery Technology and Novel Formulation, Institute of Materia Medica, Chinese Academy of Medical Sciences and Peking Union Medical College, Beijing, China

**Keywords:** *morus alba* L. (Sangzhi) alkaloids, type 2 diabetes, islet β cells, insulin secretion, apoptosis, dedifferentiation

## Abstract

**Background:** *Morus alba* L. (Sangzhi) alkaloids (SZ-A), extracted from the Chinese herb *Morus alba* L. (mulberry twig), have been shown to ameliorate hyperglycemia in type 2 diabetes and have been approved for diabetes treatment in the clinic. However, their versatile pharmacologic effects and regulatory mechanisms are not yet completely understood.

**Purpose:** This study explored the protective effects of SZ-A on islet β cells and the underlying mechanism.

**Methods:** Type 2 diabetic KKA^y^ mice were orally administered SZ-A (100 or 200 mg/kg, once daily) for 11 weeks, and oral glucose tolerance, insulin tolerance, intraperitoneal glucose tolerance and hyperglycemia clamp tests were carried out to evaluate the potency of SZ-A *in vivo*. The morphology and β-cell dedifferentiation marker of KKA^y^ mouse islets were detected via immunofluorescence. The effect of SZ-A on glucose-stimulated insulin secretion was investigated in both the islet β-cell line MIN6 and mouse primary islets. Potential regulatory signals and pathways in insulin secretion were explored, and cell proliferation assays and apoptosis TUNEL staining were performed on SZ-A-treated MIN6 cells.

**Results:** SZ-A alleviated hyperglycemia and glucose intolerance in type 2 diabetic KKA^y^ mice and improved the function and morphology of diabetic islets. In both MIN6 cells and primary islets, SZ-A promoted insulin secretion. At a normal glucose level, SZ-A decreased AMPKα phosphorylation, and at high glucose, SZ-A augmented the cytosolic calcium concentration. Additionally, SZ-A downregulated the β-cell dedifferentiation marker ALDH1A3 and upregulated β-cell identifying genes, such as *Ins1*, *Ins2*, *Nkx2.2* and *Pax4* in KKA^y^ mice islets. At the same time, SZ-A attenuated glucolipotoxicity-induced apoptosis in MIN6 cells, and inhibited Erk1/2 phosphorylation and caspase 3 activity. The major active fractions of SZ-A, namely DNJ, FAG and DAB, participated in the above regulatory effects.

**Conclusion:** Our findings suggest that SZ-A promotes insulin secretion in islet β cells and ameliorates β-cell dysfunction and mass reduction under diabetic conditions both *in vivo* and *in vitro*, providing additional supportive evidence for the clinical application of SZ-A.

## Introduction

Type 2 diabetes is a great threat to human health worldwide. In diabetic patients, because of hyperglycemia, many chronic complications, such as angiocardiopathy, retinopathy, nephropathy, and neuropathy, often occur. Progressive reductions in β-cell function and mass comprise the central pathogenic mechanism of type 2 diabetes. Although many therapeutics have been deployed to combat hyperglycemia, few of them directly target β-cell pathogenesis besides of GLP-1 analogs, such as liraglutide (Santilli et al., 2017), and dipeptidyl peptidase-4 (DPP-4) inhibitors, such as sitagliptin (Xu et al., 2008).

The progression of β-cell dysfunction includes not only defective insulin secretion but also β-cell mass reduction, which results from β-cell apoptosis, the failure of existing β cells to proliferate, and β-cell dedifferentiation under metabolic stress such as hyperglycemia, hyperlipidemia and chronic inflammation (Sun and Han, 2020; Ying et al., 2020; Amo-Shiinoki et al., 2021). However, given the reversibility of β cells, the reduction in β-cell mass should not be completely ascribed to apoptosis (Marselli et al., 2014). Recently, increasing evidence has suggested that β-cell failure may be mainly due to increased dedifferentiation (Cinti et al., 2016; Ishida et al., 2017; Sun et al., 2019). Therefore, preventing dedifferentiation or promoting β-cell redifferentiation after the occurrence of dedifferentiation may be another method for type 2 diabetes therapy.

The traditional Chinese medicine *Morus alba* L. (Sangzhi) alkaloids (SZ-A) tablets have been approved by the China National Medical Products Administration for type 2 diabetes mellitus (T2DM) treatment in China. Qu et al. (Qu et al., 2021) demonstrated that SZ-A tablets possess effective hypoglycemic effects with few adverse events, suggesting good safety in clinical trials. SZ-A contains three major effective fractions, namely, 1-deoxynojirimycin (DNJ), 1,4-dideoxy-1,4-imino-D-arabinitol (DAB) and fagomine (FAG) (Yang et al., 2015; Yang et al., 2017), which are extracts from the Chinese herb *Morus alba* L. (mulberry twig), the dried young branch of *Morus alba* L. SZ-A not only inhibits the activity of α-glucosidases, especially sucrase and maltase, *in vitro* (Liu et al., 2019) but also ameliorates intestinal flora imbalance and hyperglycemia in diabetic KKA^y^ mice (Liu et al., 2015; Liu et al., 2021; Yuling). However, the protective effect and mechanism of SZ-A on β-cell function in the progression of diabetes remain to be further identified.

Our observations support a novel role for SZ-A in β-cell function and mass. We show that SZ-A may act on more targets than glucosidase. It not only promotes β-cell insulin secretion but also ameliorates β-cell loss by preventing β-cell dedifferentiation and apoptosis.

## Material and Methods

### Drug and Reagents

1-Deoxynojirimycin (DNJ) and fagomine (FAG) were purchased from MedChem Express (HY-14860 and HY-13005, USA). 1,4-Dideoxy-1,4-imino-D-arabinitol (DAB) was purchased from Sigma (D1542, USA). *Morus alba* L. (Sangzhi) alkaloid (SZ-A) powder (Lot number: 201708008; the total polyhydroxy alkaloids content in SZ-A powder is approximately 63%, which includes 39% DNJ, 10.5% FAG, and 7% DAB, as shown in Supplementary Figure S1) was synthesized and provided by Beijing Wehand-bio Pharmaceutical Co. Ltd. (Beijing, China).

### Animal Experimental Design

**FIGURE 1:**
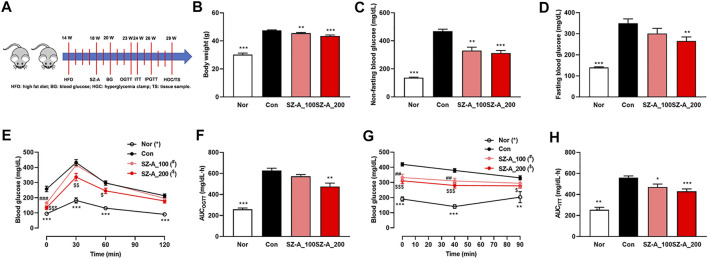
SZ-A alleviated hyperglycemia and glucose intolerance in type 2 diabetic KKA^y^ mice. KKA^y^ mice were treated with 100 or 200 mg/kg SZ-A or vehicle orally once per day (*n* = 10 mice per group). **(A)** Timeline of the animal experiment. **(B)** Body weight. **(C)** Non-fasting blood glucose levels. **(D)** Fasting blood glucose. **(E)** Oral glucose tolerance test (OGTT) and **(F)** area under the curve (AUC) of the OGTT. **(G)** Insulin tolerance test (ITT) and **(H)** area under the curve (AUC) of the ITT. All data are expressed as the mean ± SEM. *p < 0.05, **p < 0.01, ***p < 0.001, vs. the Con group.

C57BL/6J mice (male, 14 weeks) were fed a normal diet and comprised the normal group (Nor group). KKA^y^ mice (male, 14 weeks) were fed high-fat diets (45% of kcal from fat; D12451; Research Diets, USA). After 4 weeks, the KKA^y^ mice were randomly divided into three groups (Con group and two doses of SZ-A-treated groups, 10 mice per group) according to the levels of fasting blood glucose, triglycerides, total cholesterol, body weight and the percentage of increasing blood glucose level at 30 min after glucose (2.0 g/kg) overloading (Diagram of animal experiment was shown as Figure 1A). The SZ-A groups were orally administered SZ-A (100 mg/kg, SZ-A_100; 200 mg/kg, SZ-A_200) once per day. The Nor group and Con group were treated with vehicle. All animals were housed in communal cages at 23 ± 1°C on a 12-h light-dark cycle with free access to food and water. Blood glucose was tested in the 2nd week. The oral glucose tolerance test (OGTT), insulin tolerance test (ITT) and intraperitoneal glucose-stimulated insulin secretion test (IPGSIST) were carried out consecutively on the 5th, 6th, and 8th weeks. The hyperglycemia clamp test was conducted in the 11th week. Then, the mice were sacrificed *via* cervical dislocation, and the pancreas was separated and subsequently fixed with formalin. The experiments were conducted following the “3R” principles and the standards for laboratory animals (GB14925-2001 and MOST 2006a) established by the People’s Republic of China. The animal protocol was approved by the Institutional Animal Care and Use Committee of the Institute of Materia Medica, Chinese Academy of Medical Sciences and Peking Union Medical College (Beijing, China).

### OGTT, ITT and IPGSIST

The mice were fasted for 6 h, and blood was obtained from the tail for the fasting blood glucose test. For the OGTT, blood samples were acquired at 0, 30, 60 and 120 min after oral glucose overload (2.0 g/kg) for the blood glucose test. For the ITT, mice were hypodermically injected with insulin (0.4 IU/kg), and subsequently, blood was taken from the tail at 40 and 90 min. For the IPGSIST, blood samples were acquired at 0 and 30 min after intraperitoneal injection of glucose (2.0 g/kg) for blood glucose and insulin tests.

### The Hyperglycemic Clamp Test

The mice were anesthetized with amobarbital sodium after fasting overnight, and then 5% (*w/v*) glucose was perfused into the jugular vein through a peristaltic pump. During the first 1 min, a glucose bolus (100 mg/kg) was given. Blood was obtained from the tail at 0, 2, 5, 10, and 15 min after glucose loading for the phase I insulin secretion test. Subsequently, glucose was microperfused persistently until the blood glucose level remained steady at 14.0 ± 0.5 mM. The whole testing process took approximately 2.0–2.5 h, during which blood was obtained at 60, 100, and 120 min for the phase II insulin secretion test.

### Morphology and Immunohistochemistry of Islets From KKA^y^ Mice Treated With SZ-A

The fixed pancreases of KKA^y^ mice were embedded in paraffin. Tissues were sliced to 5 μm for morphology analyses. Some sections were stained with hematoxylin-eosin to observe the morphology of islets under a microscope. Others were immunostained with rabbit anti-glucagon antibody (1:200, ab92517, Abcam, USA), rabbit anti-ALDH1A3 antibody (1:100, NBP2-15339, NOVUS, USA), and rat anti-insulin antibody (1:200, MAB1417, R&D Systems, USA). The secondary antibodies were Alexa Fluor 488 donkey anti-rabbit IgG (1:200, A-21206, Invitrogen, USA) and Alexa Fluor 594 donkey anti-rat IgG (1:200, A-11007, Invitrogen, USA). Then, the tissues were mounted utilizing mounting medium with DAPI (ZLI-9557, ZSGB-BIO, China). Finally, the tissues were visualized by confocal laser scanning microscopy (CLSM) (Leica Microsystems, Germany). ImageJ software was used to analyze the picture. The area percentage of β-cell/islet and α-cell/islet were calculated via the insulin-positive or glucagon-positive area in one islet divided by the islet area. The ratio of ALDH1A3-positive β cells/β cells were calculated via the both ALDH1A3 and insulin-positive area divided by insulin-positive area in the islet.

### Cells and Primary Islet Preparation

The mouse β-cell line MIN6 was a gift from Professor Xiao Han at Nanjing Medical University, and the cells were cultured in Dulbecco’s modified Eagle’s medium (DMEM, 25 mM glucose) supplemented with 15% fetal bovine serum (FBS) (Chen et al., 2016). Mouse islets were isolated from C57BL/6J mice (male, 24 ± 2 g) and diabetic KKA^y^ mouse model (male, 38 ± 2 g) using the collagenase V (C9263, Sigma, Germany) perfusion method and were cultured in RPMI-1640 medium supplemented with 10% FBS as reported previously (Lei et al., 2015).

### Glucose-Stimulated Insulin Secretion Assay

MIN6 cells and mouse islets were prepared to test insulin secretion in response to low and high concentrations of glucose. MIN6 cells were plated in 96-well plates (5 × 10^4^/well) and starved for 1 h in Krebs buffer (2.8 mM glucose) with or without different concentrations of SZ-A, DNJ, FAG or DAB. Then, the cells were cultured for another 1 h in new Krebs buffer containing 2.8 mM or 16.8 mM glucose combined with SZ-A, DNJ, FAG, DAB or vehicle. Ten islets were transferred to new Krebs buffer containing 2.8 or 16.8 mM glucose and were cultured for 1 h after fasting for 1 h in Krebs buffer with 2.8 mM glucose, which was combined with SZ-A, DNJ, FAG, DAB or vehicle. The supernatant was collected for the insulin test using the Mouse Ultrasensitive Insulin ELISA Kit (80-INSMSU-E10, ALPCO, USA). MIN6 cells and mouse islets were used for quantitative protein determination with BCA reagents (P1511, APPLYGEN, China).

### Estimation of the Free Cytosolic Ca^2+^ Concentration

MIN6 cells in 96-well plates were cultured with 25, 50, and 100 μg/ml SZ-A; 10 μg/ml FAG; 10 μg/ml DAB; or vehicle for 1 h. The cells were then loaded with the Ca^2+^-sensing fluorescent probe Fluo-4 AM (F14201, Invitrogen, USA) for 30 min at 37°C. The internal calcium concentration ([Ca^2+^]_i_) was measured using a Multimode Microplate Reader (Synergy 2, BIO-TEK, USA) through time-series recording of the fluorescent signal. A 5-min basal recording was acquired when the cells were in Krebs buffer containing 2.8 mM glucose. For stimulation, the cells were perfused with Krebs buffer containing elevated glucose concentration (16.8 mM) combined with SZ-A or vehicle. The changes in the MIN6 cell [Ca^2+^]_i_ were recorded for 20 min after stimulation.

### Measurement of cAMP Produced in MIN6 Cells

Intracellular cAMP ([cAMP]_i_)was measured with a LANCE cAMP Detection Kit (AD0262, PerkinElmer, USA) through time-resolved fluorescence resonance energy transfer (TR-FRET) technology. MIN6 cells were preincubated for 1 h in Krebs buffer containing 2.8 mM glucose and different concentrations of SZ-A or vehicle. Then, both Alexa Fluor 647-cAMP antibody solution and stimulation solution containing 25 μM isobutyl methylxanthine (312460, J&K, China) and different concentrations of SZ-A or vehicle were added to each well and cultured for 30 min. The europium chelate of the Eu-SA/b-cAMP tracer was subsequently added to test the cAMP production of MIN6 cells through a multilabel reader (Envision 2104, PerkinElmer, USA).

### High Glucose and Palmitic Acid Treatment

For high glucose (Glc) and palmitic acid (PA) treatment, MIN6 cells were incubated in DMEM with 15% FBS, 33 mM glucose and 0.25 mM palmitic acid. The blank control cells were incubated in DMEM with 15% FBS, 25 mM glucose and 8 mM mannitol (63559, Sigma, USA).

### Cell Proliferation Assay

MIN6 cells were seeded at 3 × 10^4^/100 μl per well in 96-well plates and incubated under normal conditions. The next day, they were treated with 25, 50, or 100 μg/ml SZ-A or vehicle for 24 h. At the same time, high glucose- and palmitic acid-treated MIN6 cells were co-incubated with different concentrations of SZ-A, DNJ, FAG, DAB or vehicle for 24 h. Cell proliferation was detected by EdU labeling according to the recommended protocol of the assay kit (C0071, Beyotime, China). The cells were fixed with 4% paraformaldehyde, and the nuclei were labeled with Hoechst 33342. The fluorescence signal was detected at 530/25 nm (EdU signal) and 460/40 nm (Hoechst 33342 signal) with a multifunctional microplate reader (Synergy 2, BioTek, USA) and calculated as the ratio of Edu to Hoechst 33342.

### TUNEL Assay

High glucose- and palmitic acid-treated MIN6 cells were coincubated with different concentrations of SZ-A, DNJ, FAG, DAB or vehicle for 72 h and fixed with 4% paraformaldehyde. The TUNEL assay was performed according to the recommended procedure of the *TransDetect* ^®^ *In Situ* Fluorescein TUNEL Cell Apoptosis Detection Kit (FA201-01, TransGen Biotech, China). Finally, the cells were mounted utilizing mounting medium with DAPI (ZLI-9557, ZSGB-BIO, China). The images were visualized by inverted fluorescence microscopy (Olympus, Japan). The data are presented as the ratio of TUNEL/DAPI.

### Caspase 3 Activity Assay

High glucose- and palmitic acid-treated MIN6 cells were coincubated with different concentrations of SZ-A, DNJ or vehicle for 24 h. Then, the activity of caspase 3 was tested using Caspase-Glo®3/7 Assays (G8981, Promega, USA). The signal was detected with a multifunctional microplate reader (Synergy 2, BioTek, USA).

### Western Blot Analysis

The islets of KKA^y^ mice were incubated in RPMI-1640 medium containing vehicle or 100 μg/ml SZ-A for 24 h. MIN6 cells were fasted for 1 h in Krebs buffer (2.8 mM glucose) with or without different concentrations of SZ-A, FAG, or DAB. Then, they were cultured for another 1 h in new Krebs buffer containing 2.8 mM or 16.8 mM glucose combined with SZ-A, FAG, DAB or vehicle. High glucose- and palmitic acid-treated MIN6 cells were coincubated with different concentrations of SZ-A, DNJ or vehicle for 24 h.

Islets or MIN6 cells were homogenized in RIPA lysis buffer (C1053; APPLYGEN, China) containing protease inhibitors (P1265; APPLYGEN, China) and phosphatase inhibitors (P1260; APPLYGEN, China). Protein (10 μg) from each sample was resolved by sodium dodecyl sulfate–polyacrylamide gel electrophoresis and transferred to polyvinylidene fluoride membranes for immunoblotting. Rabbit anti-phospho-AMPKα (1:1000, 2535s), rabbit anti-AMPKα (1:1000, 2532s), rabbit-phospho-Erk (1:1000, 9101s), rabbit anti-Erk (1:1000, 9102s), and rabbit anti-HSP90 (1:1000, 4877s) were purchased from Cell Signaling Technology (USA). Goat anti-rabbit IgG/HRP (1:5000, ZDR-5306) was purchased from ZSGB-BIO (China).

### Quantitative PCR

The islets of KKA^y^ mice were incubated in RPMI-1640 medium containing vehicle or 100 μg/ml SZ-A for 24 h. RNA was isolated using TRIzol Reagent (15596018, Life Technology, USA), and cDNA was synthesized using *TransScript* ^®^ One-Step gDNA Removal and cDNA Synthesis SuperMix (AT311, TransGen Biotech, China) for gene expression analysis. Quantitative PCR (qPCR) was performed with TransStart Tip Green qPCR SuperMix (AQ141–04, TransGen Biotech, China) using the primers shown in Table 1.

**TABLE 1:** The primer list of genes for qPCR.

Gene	Forward primer	Reverse primer
*Aldh1a3* (NM_053080.3)	5′-GAG​ACC​CCT​TCG​ATG​CCA​AA-3′	5′-CCG​TGG​GTT​TGA​TGA​ACA​GC-3′
*Ins1* (NM_008386.4)	5′-CTG​GTG​GGC​ATC​CAG​TAA​CC-3′	5′-CAA​AAG​CCT​GGG​TGG​GTT​TG-3′
*Ins2* (NM_001185083.2)	5′-CCA​TCA​GCA​AGC​AGG​AAG​GTT​A-3′	5′-GCT​TGA​CAA​AAG​CCT​GGG​TG-3′
*Pdx1* (NM_008814.3)	5′-AGC​GTT​CCA​ATA​CGG​ACC​AG-3′	5′-TGC​TCA​GCC​GTT​CTG​TTT​CT-3′
*NeuroD* (NM_010894.2)	5′-CCC​TAC​TCC​TAC​CAG​TCC​CC-3′	5′-GAG​GGG​TCC​GTC​AAA​GGA​AG-3′
*Nkx6.1* (NM_144955.2)	5′-GGC​TGT​GGG​ATG​TTA​GCT​GT-3′	5′-TCATCTC GGCCATACTGTGC-3′
*Pc1* (NM_013628.2)	5′-CTT​GCT​TCT​TTT​CTC​CCA​GCC-3′	5′-ACC​AAA​CGC​AAA​AGA​AGG​CG-3′
*Pc2* (NM_008792.4)	5′-TTT​GGA​GTC​CGA​AAG​CTC​CC-3′	5′-GGT​GTA​GGC​TGC​GTC​TTC​TT-3′
*MafA* (NM_194350.1)	5′-CCA​GCT​GGT​ATC​CAT​GTC​CG-3′	5′-CTC​TGG​AGC​TGG​CAC​TTC​TC-3′
*Gcg* (NM_008100.4)	5′-CAG​AAG​AAG​TCG​CCA​TTG​CC-3′	5′-GAA​GTC​CCT​GGT​GGC​AAG​AT-3′
*MafB* (NM_010658.3)	5′-CAA​CGG​TAG​TGT​GGA​GGA​CC-3′	5′-CTT​CTG​CTT​CAG​GCG​GAT​CA-3′
*Pax6* (NM_001244198.2)	5′-CCG​AGA​AGC​GGC​TTT​GAG​AA-3′	5′-TCA​CCG​CCC​TTG​GTT​AAA​GT-3′
*actin* (NM_009609.3)	5′-ACT​CTT​CCA​GCC​TTC​CTT​C-3′	5′-ATC​TCC​TTC​TGC​ATC​CTG​TC-3′

### Statistical Analysis

GraphPad Prism 8 was used for all statistical analyses. Two experimental groups were analyzed by Student’s *t* test. Multiple groups were compared by one-way analysis of variance (ANOVA) followed by Dunnett’s *t* test or two-way ANOVA with Tukey’s test depending on the experiments. Differences were considered statistically significant at *p* < 0.05.

## Results

### SZ-A Alleviates Hyperglycemia and Glucose Intolerance in Type 2 Diabetic KKA^y^ Mice

The type 2 diabetic KKA^y^ mice have a similar metabolic syndrome phenotype as humans and exhibit hyperglycemia, hyperinsulinemia, hyperlipidemia and obesity. We have reported that SZ-A ameliorates glucose and lipid metabolism in KKA^y^ mice (Liu et al., 2021). In this study, we again identified that non-fasting and fasting blood glucose was reduced significantly with SZ-A treatment (Figures 1C,D), which may be ascribed to the increased insulin secretion and sensitivity. In order to identify these further, OGTT, ITT and IPGSIST were carried out sequentially. The OGTT and ITT showed that SZ-A alleviated hyperglycemia after oral glucose load (Figures 1E,F) and IP injection of insulin (Figures 1G,H) in a dose-dependent manner. Notably, 200 mg/kg SZ-A could ameliorate glucose tolerance significantly. At the same time, body weight in the two SZ-A treatment groups decreased significantly (Figure 1B).

### SZ-A Amends Islet β-Cell Dysfunction in Type 2 Diabetic KKA^y^ Mice

**FIGURE 2:**
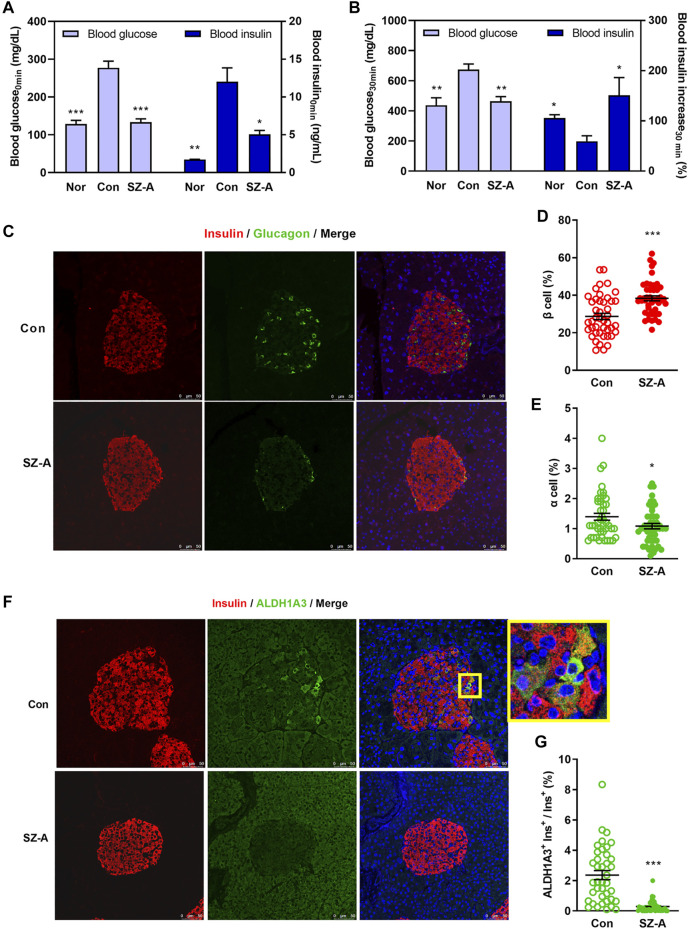
SZ-A amended β-cell dysfunction in type 2 diabetic KKA^y^ mice. **(A,B)** An intraperitoneal glucose-stimulated insulin secretion test (IPGSIST) was executed in KKA^y^ mice after treating with SZ-A (200 mg/kg) for 8 weeks (*n* = 5 mice per group). **(A)** Blood glucose and insulin levels at 0 min before injection of glucose. **(B)** Blood glucose and the percentage of increased blood insulin at 30 min after glucose (2.0 g/kg) load. **(C–G)** Immunohistochemistry of the pancreas of KKA^y^ mice in the Con group and SZ-A (200 mg/kg) group (*n* = 5 mice per group). β Cells were labeled with insulin antibody (red), α cells were labeled with glucagon antibody (green), dedifferentiated cells were labeled with ALDH1A3 antibody (green). **(D)** The percentage of β-cell/islet area; **(E)** the percentage of α-cell/islet area; and **(G)** the ratio of ALDH1A3-positive β cells/β cells. All data are expressed as the mean ± SEM. *p < 0.05, **p < 0.01, ***p < 0.001, vs. the Con group.

IPGSIST was carried out in KKA^y^ mice. We observed that at either 0 min or 30 min after IP injection of glucose (2.0 g/kg), SZ-A significantly decreased blood glucose (Figures 2A,B). In addition, blood insulin at 0 min was significantly reduced by SZ-A, but insulin secretion after glucose overload was promoted significantly in the SZ-A group (Figures 2A,B). Subsequently, the hyperglycemic clamp test showed that SZ-A could raise the first-phase insulin secretory response to glucose by 86.17% (AUC_0–15 min_) compared with that of the Con group (Supplementary Figures S2B,C).

The morphology of islets was depicted by staining glucagon (green) in α cells and insulin (red) in β cells. The islet architecture of the Con group was damaged, as evidenced by the slightly diminished β cells as well as by the augmented and diffusely distributed α cells. Despite all of these changes, in the SZ-A treated islets, more β cells were interspersed among the islets, and α cells tended to be located toward the edge of the islets (Figure 2C), which were associated with significantly increased β-cell area and decreased α-cell area (Figures 2D,E). At the same time, the ALDH1A3^+^ cells were significantly diminished in the SZ-A group (Figures 2F,G). Moreover, some cells in the central area of the islets displayed vacuolar degeneration and necrosis in mice of the Con group, but the islet morphology in the SZ-A group was normal (Supplementary Figure S2A).

### SZ-A Stimulates Insulin Secretion in the Mouse β-Cell Line MIN6 and Mouse Primary Islets

**FIGURE 3:**
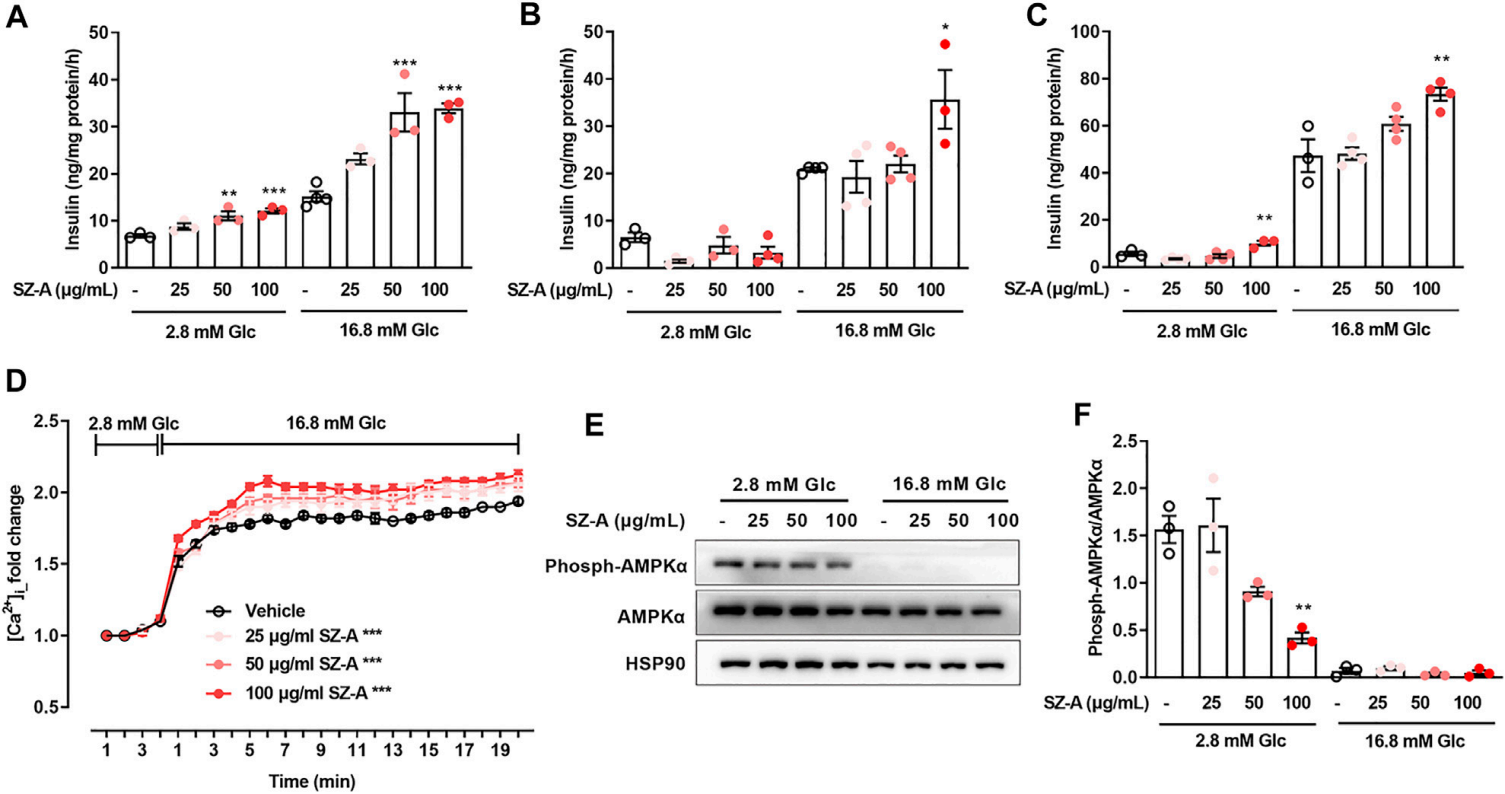
SZ-A promoted insulin secretion in MIN6 cells and mouse primary islets, and advanced glucose-dependent [Ca^2+^]_i_ changes and inhibited AMPKα phosphorylation in MIN6 cells. **(A–C)** MIN6 cells or islets were preincubated with 25, 50, or 100 μg/ml SZ-A or vehicle for 1 h. Glucose-stimulated insulin secretion assay in MIN6 cells **(A)**, islets of normal C57BL/6J mice **(B)** and islets of type 2 diabetic KKA^y^ mice **(C)** at glucose concentrations of 2.8 and 16.8 mM (*n* = 3 or 4 replicates per group). **(D)** The changes in [Ca^2+^]_i_ of MIN6 cells were labeled by Fluo4-AM when MIN6 cells were treated with 25, 50, or 100 μg/ml SZ-A or vehicle while elevating the glucose concentration from 2.8 to 16.8 mM (*n* = 5 replicates in each condition). **(E,F)** AMPKα expression level and AMPKα phosphorylation level in MIN6 cells incubated with 25, 50, or 100 μg/ml SZ-A or vehicle at glucose concentrations of 2.8 and 16.8 mM for 1 h (*n* = 3 replicates in each group). All data are expressed as the mean ± SEM. *p < 0.05, **p < 0.01, ***p < 0.001, vs. the vehicle group.

To investigate the possible role of SZ-A in the β-cells insulin secretion, both the mouse β-cell line MIN6 and mouse primary islets were treated with SZ-A. In both MIN6 cells (Figure 3A) and KKA^y^ mouse islets (Figure 3C), SZ-A at 100 μg/ml promoted insulin secretion regardless of the glucose concentration (2.8 mM or 16.8 mM). In C57BL/6J mouse islets, SZ-A mainly enhanced insulin secretion at 16.8 mM glucose (Figure 3B). In addition, three major effective fractions, namely, DNJ, FAG and DAB, showed different efficacies on insulin secretion in β cells. Among them, DAB and FAG promoted insulin secretion in MIN6 cells, but DNJ did not (Supplementary Figure S3A). In addition, DAB and FAG facilitated insulin secretion in C57BL/6J mouse islets and KKA^y^ mouse islets, respectively (Supplementary Figures S3B,C). However, SZ-A has a greater ability to stimulate insulin secretion in β cells than any of its constituents.

### Increased [Ca^2+^]_i_ and Decreased AMPKα Phosphorylation Are Involved in Insulinotropic Effect of SZ-A in MIN6 Cells

Glucose facilitates insulin secretion through changes in the free [Ca^2+^]_i_ and [cAMP]_i_, so we examined the actions of SZ-A on [Ca^2+^]_i_ and [cAMP]_i_ in MIN6 cells. In MIN6 cells, elevating the glucose concentration from 2.8 to 16.8 mM initiated a marked rise in [Ca^2+^]_i_ and [cAMP]_i_. However, in the presence of 2.8 mM glucose, SZ-A had no effect on [Ca^2+^]_i_. At a glucose concentration of 16.8 mM, changes in [Ca^2+^]_i_ were observed after SZ-A treatment (Figure 3D). At the same time, 100 μg/ml SZ-A significantly decreased AMPK phosphorylation levels (Figures 3E,F) at 2.8 mM glucose. However, in the presence of 2.8 mM or 16.8 mM glucose, SZ-A did not cause an increase in [cAMP]_i_ (Supplementary Figure S4A). In addition, two of the major effective fractions, that is, FAG and DAB, modulated the glucose-dependent increases in [Ca^2+^]_i_ (Supplementary Figure S4B) and inhibited AMPK phosphorylation at 2.8 mM glucose (Supplementary Figure S4C).

### SZ-A Ameliorates β-Cell Dedifferentiation in Type 2 Diabetic KKA^y^ Mouse Islets

**FIGURE 4:**
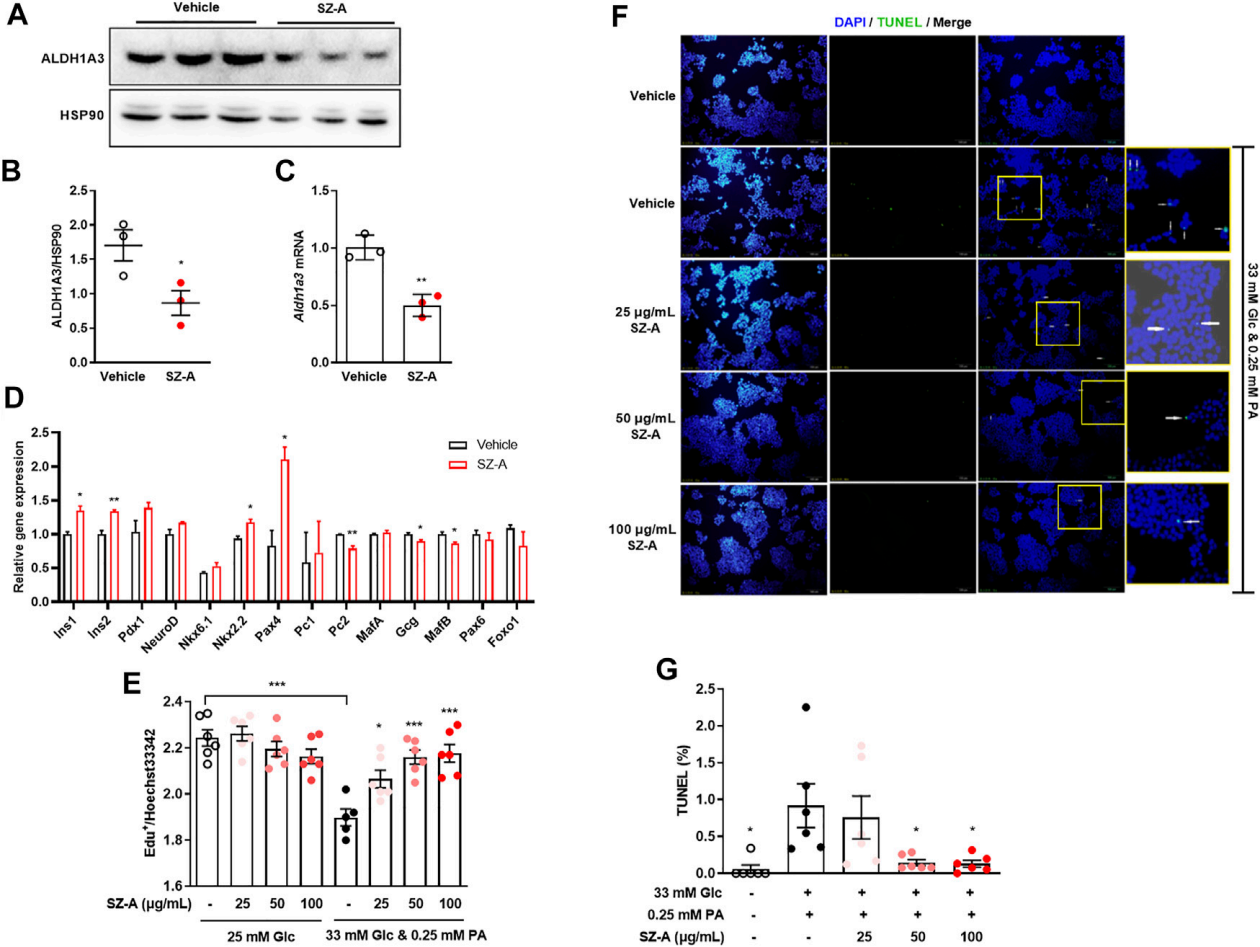
SZ-A prevented β-cell dedifferentiation in type 2 diabetic KKA^y^ mouse islets, and apoptosis in high glucose- and PA-treated MIN6 cells. **(A,B)** ALDH1A3 expression levels, **(C)** *Aldh1a3* mRNA levels and **(D)** β-cell and α-cell relative gene expression levels in KKA^y^ mouse islets treated with 100 μg/ml SZ-A or vehicle for 24 h (*n* = 3 replicates in each group). All data are expressed as the mean ± SEM. *p < 0.05, **p < 0.01, vs. the vehicle group. **(E)** MIN6 cells were treated with high glucose and PA and 25, 50, or 100 μg/ml SZ-A or vehicle for 24 h. The EdU assay was performed in MIN6 cells (*n* = 6 replicates per group). **(F,G)** MIN6 cells were treated under the same conditions for 72 h. **(F)** Nuclei were labeled with DAPI (blue), and apoptotic bodies were marked by TUNEL (green). **(G)** The ratio of TUNEL-positive cells/cell nucleus. All data are expressed as the mean ± SEM (*n* = 5–6 replicates per group). *p < 0.05, ***p < 0.001, vs. the Glc + PA group.

Islet β-cell dedifferentiation has been suggested to participate to the β-cell failure (Zhang and Liu, 2020), and Aldh1a3 was reported as a marker of β-cell dedifferentiation (Kim-Muller et al., 2016). In KKA^y^ mice, the Aldh1a3 expression level was significantly increased in islet β cells compared with normal mice (Supplementary Figure S5). The expression level of Aldh1a3 was inhibited significantly in KKA^y^ mouse islets treated with SZ-A (Figures 4A–C). Moreover, in islet cells, SZ-A increased β-cell identifying genes such as *Ins1*, *Ins2*, *Nkx2.2* and *Pax4* and decreased β-cell excluding genes such as *Gcg* and *MafB* (Figure 4D).

### SZ-A Attenuates Apoptosis, Accompanied With Downregulated Erk1/2 Phosphorylation and Caspase 3 Activity in High Glucose- and Palmitic Acid-Treated MIN6 Cells

In addition to dedifferentiation, apoptosis was another cause to β-cell mass reduction. High glucose and palmitic acid treatment (24 h) inhibited proliferation, and if treatment was prolonged to 72 h, apoptosis were recognized by increased TUNEL-positive cells. SZ-A did not influence cell proliferation in basic culture medium (25 mM glucose) but significantly reversed the growth inhibition of MIN6 cells induced by high glucose and palmitic acid (Figure 4E). Furthermore, SZ-A at concentrations of 50 and 100 μg/ml significantly decreased the number of TUNEL-positive cells after 72 h of high glucose and palmitic acid treatment (Figures 4F,G). Interestingly, among the three major fractions of SZ-A, DNJ but not FAG and DAB showed a similar effect (Supplementary Figure S6).

**FIGURE 5:**
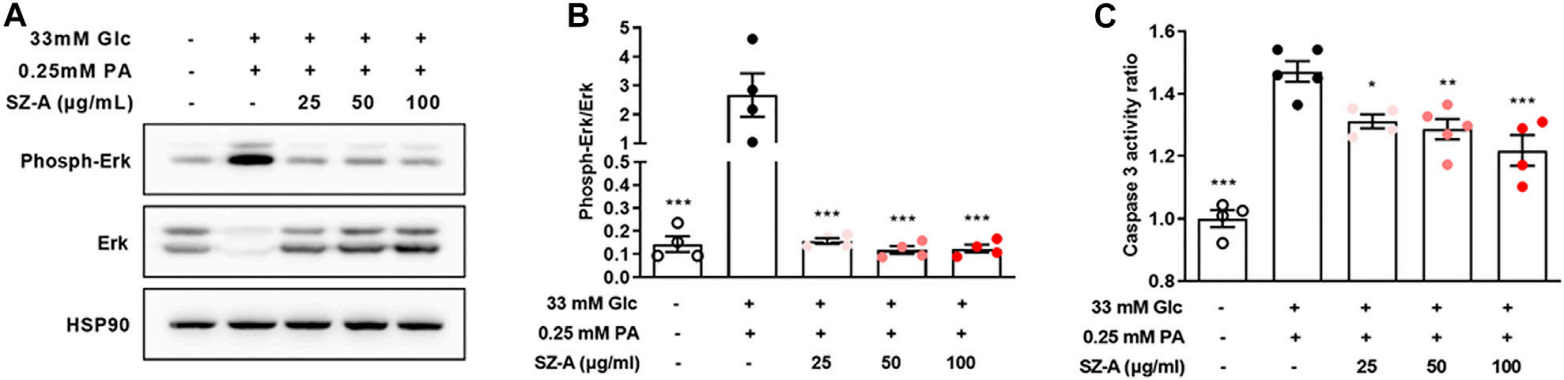
SZ-A decreased Erk1/2 phosphorylation and caspase 3 activity in high glucose- and PA-treated MIN6 cells. MIN6 cells were treated with high glucose and PA and 25, 50, or 100 μg/ml SZ-A or vehicle for 24 h. **(A,B)** Erk1/2 expression levels and Erk1/2 phosphorylation levels in MIN6 cells (*n* = 4 replicates in each condition). **(C)** Caspase 3 activity ratio in MIN6 cells (*n* = 4–5 replicates in each condition). *p < 0.05, **p < 0.01, ***p < 0.001, vs. the Glc + PA group.

Furthermore, SZ-A significantly attenuated the elevated Erk1/2 phosphorylation level and caspase 3 activity under high glucose- and palmitic acid-induced stress conditions (Figures 5A–C). Similarly, DNJ, one of the major fractions of SZ-A, decreased both Erk1/2 phosphorylation and caspase 3 activity (Supplementary Figure S7).

## Discussion

SZ-A, as a natural medicine with effective components (no less than 50% alkaloids), was approved as a clinical therapeutic for type 2 diabetic patients in 2020 in China. As reported in a phase IIIa clinical study, SZ-A can ameliorate hyperglycemia and reduce HbA1c in type 2 diabetic patients (Qu et al., 2021). In addition, in our previous study, we showed that SZ-A maintains the intestinal flora balance and promotes GLP-1 secretion (Liu et al., 2021). Given that β-cell failure is related to the transition from prediabetes to diabetes, improved β-cell function has a major role in preventing type 2 diabetes. Our previous research pointed out that SZ-A may ameliorate β-cell dysfunction (Liu et al., 2021). However, the substantial effects and underlying mechanism of SZ-A on islet β-cell function need to be identified further. Herein, we demonstrate that SZ-A not only promotes insulin secretion in β cells but also preserves β cells mass by preventing β-cell dedifferentiation and apoptosis.

Many studies have provided evidence that changes in the cytoplasmic concentrations of calcium and/or cAMP influence insulin secretion by β cells (Lei et al., 2015; Tengholm and Gylfe, 2017). Similarly, we found that SZ-A could augment the glucose-dependent changes in the [Ca^2+^]_i_ of β cells but had no effect on the [cAMP]_i_, which suggests that SZ-A-induced increases in [Ca^2+^]_i_ and that improvements in Ca^2+^-dependent exocytosis may be the possible mechanism by which it promotes glucose-stimulated insulin secretion. Recently, the biological role of AMPK in β cells has garnered considerable interest. Indeed, as blood glucose concentrations rise from fasting levels to feeding levels, the AMPK phosphorylation level in the islets falls (Fu et al., 2009; Swisa et al., 2015), which coincides with our results wherein the phosphorylation of AMPK was downregulated when glucose concentrations rose from 2.8 to 16.8 mM. In our research, we show that SZ-A decreases AMPK phosphorylation levels at 2.8 mM glucose but has no obvious influence at 16.8 mM glucose, which may be because the phosphorylation of AMPK has been almost completely inhibited by high glucose concentrations. The effect of SZ-A on AMPK activity may partially explain its promotion of glucose secretion at 2.8 mM glucose. However, the real mechanism by which SZ-A regulates changes in [Ca^2+^]_i_ and AMPK activity still needs to be studied further.

Hyperglycemia is an important characteristic of type 2 diabetes. In addition, hyperlipidemia is often a concomitant symptom. The concurrence of elevated glucose and fatty acid levels will cause synergistic glucolipotoxicity on islet β cells, which leads to β-cells dysfunction, including inhibition of β-cell proliferation, impaired insulin gene expression, decreased insulin secretion, dedifferentiation and apoptosis (Pascoe et al., 2012; Pan et al., 2016; Lytrivi et al., 2020).

In both animal models and patients, dedifferentiated β cells exist, and aldehyde dehydrogenase 1a3 (ALDH1A3) can be used as a dedifferentiation marker (Cinti et al., 2016; Kim-Muller et al., 2016; Sun et al., 2019; Amo-Shiinoki et al., 2021). Therefore, inducing dedifferentiated β cells to return to mature and functional β cells is a very important area of research. Some research has confirmed that dedifferentiated β cells could redifferentiate back into normal β cells, which is a drug-targetable process. Calorie restriction could prevent and reverse β-cell dedifferentiation in db/db mice (Ishida et al., 2017). Insulin supplementation during the early stage of type 2 diabetes was an effective therapy (Wang et al., 2014). Inhibition of TGF-β signaling reversed β-cell dedifferentiation in *Sel1L* ^*Ins1*^ mice (Shrestha et al., 2020). However, the therapies to reverse β-cell dedifferentiation in the clinic are still at the initial stage. Fortunately, our study showed that SZ-A dampens β-cell dedifferentiation and restores insulin-positive β cells. In addition, a subset of dedifferentiated β cells could undergo conversion into other endocrine cell types, such as Gcg^+^ cells (Talchai et al., 2012). SZ-A increases the suppression of β-cell-specific genes (*Ins1*, *Ins2*, *Nkx2.2,* and *Pax4*) but decreases the upregulation of α-cell-specific genes (*Gcg* and *MafB*) in islets of type 2 diabetic KKA^y^ mice. This demonstrates that the conversion of dedifferentiated cells to mature β cells may exist after SZ-A treatment, which possibly explains the restoration of islet β-cell function and morphology in KKA^y^ mice. However, its effect on patient islets still needs to be identified in the clinic.

In addition, β-Cell apoptosis contributes significantly to the loss of β cells in type 2 diabetes (Mandrup-Poulsen, 2001; Mathis et al., 2001). In our glucolipotoxic MIN6 cell model, SZ-A attenuated apoptosis, which may partially be explained by the increased Erk1/2 phosphorylation and caspase 3 activity induced by high glucose and palmitic acid. Erk elicits apoptosis by activating caspase 3 (Zhuang et al., 2007). Li (Li et al., 2022) also reported that mulberry leaf alkaloids and one of its active components (DNJ) could inhibit the increased apoptotic protease activity of caspase 3 in GLUTag cells. However, the ability of SZ-A to prevent β-cell apoptosis was first presented by us, and it is probably the main factor responsible for the restoration of β-cell mass and function in type 2 diabetes.

Apart from this, we also found that three major alkaloids, namely, DNJ, FAG and DAB, show different effects on the protective action of β cells. DNJ is inclined to ameliorate glucolipotoxic β-cell dysfunction, while FAG and DAB preferentially promote insulin secretion. However, the potency of each of these alkaloids is less than that of SZ-A.

## Conclusion

Our findings indicate that SZ-A, as a new type 2 diabetes therapy, promotes insulin secretion and provides more protection of β cells by preventing dedifferentiation *in vivo* and *in vitro*, and also prevents apoptosis in high glucose and palmitic acid treated insulinoma cell line. These observations offer supportive evidence and widen the clinical application of SZ-A.

## Data Availability

The original contributions presented in the study are included in the article/Supplementary Material, further inquiries can be directed to the corresponding authors.

## Abbreviations

DAB, 1,4-dideoxy-1,4-imino-D-arabinitol; DNJ, 1-deoxynojirimycin; FAG, fagomine; PA, palmitic acid; SZ-A, *Morus alba* L. (Sangzhi) alkaloids.
