# Supplementary material for: Morus alba L. (Sangzhi) Alkaloids Promote Insulin Secretion, Restore Diabetic β-Cell Function by Preventing Dedifferentiation and Apoptosis
Source: Front Pharmacol. 2022 Mar 3;13:841981. doi: 10.3389/fphar.2022.841981 (PMC8927674; doi:10.3389/fphar.2022.841981)
Supplement: Supplementary file 1 [file DataSheet1.docx]

**Morus alba L. (Sangzhi) alkaloids (SZ-A) promotes insulin secretion, restores diabetic β-cell function by preventing dedifferentiation and apoptosis**

**SUPPLEMENTARY FIGURES**


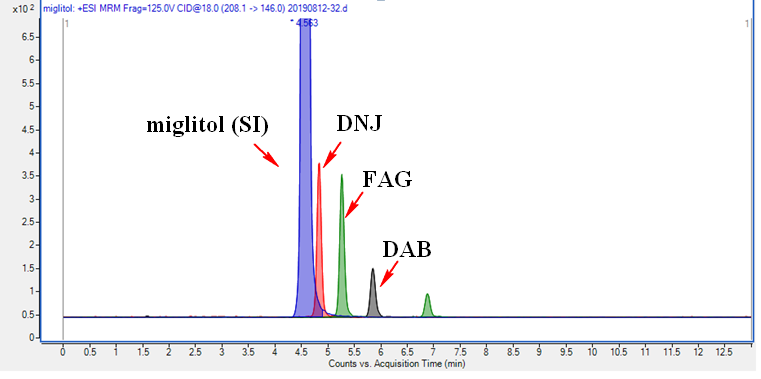


**Fig. S1.** The results of the Morus alba L. (Sangzhi) alkaloid (SZ-A) quality testing by HPLC. Miglitol is the internal standard.


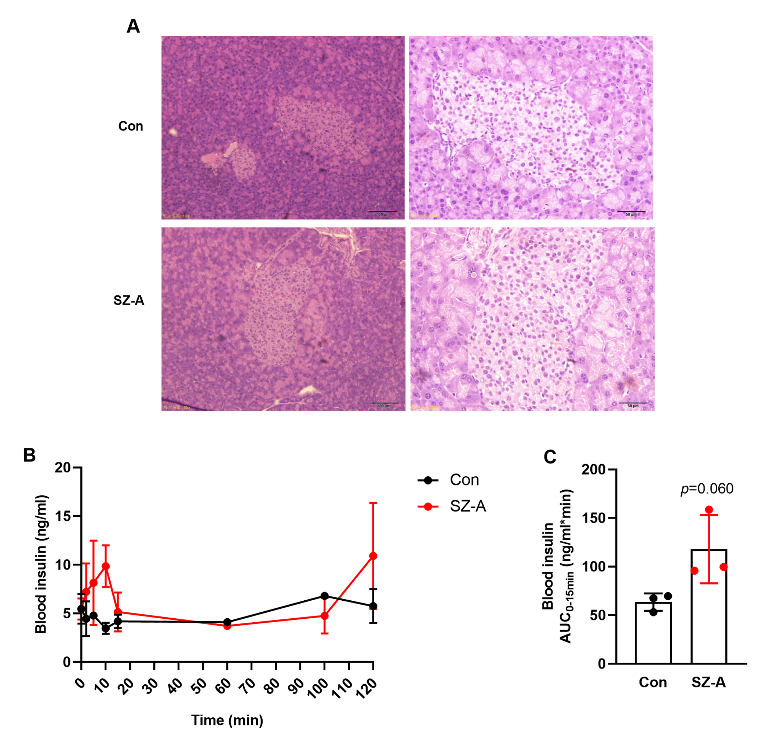


**Fig. S2.** SZ-A protected islet β cells in type 2 diabetic KKA^y^ mice. (A) Hematoxylin-eosin staining of the pancreas of KKA^y^ mice in the Con group and SZ-A group. (B) Blood insulin and (C) the area under the curve (AUC) of blood insulin during the first 15 min in the hyperglycemic clamp test in KKA^y^ mice after treating with SZ-A (200 mg/kg) for 11 weeks (n=3 mice per group). All data are expressed as the mean ± SEM.


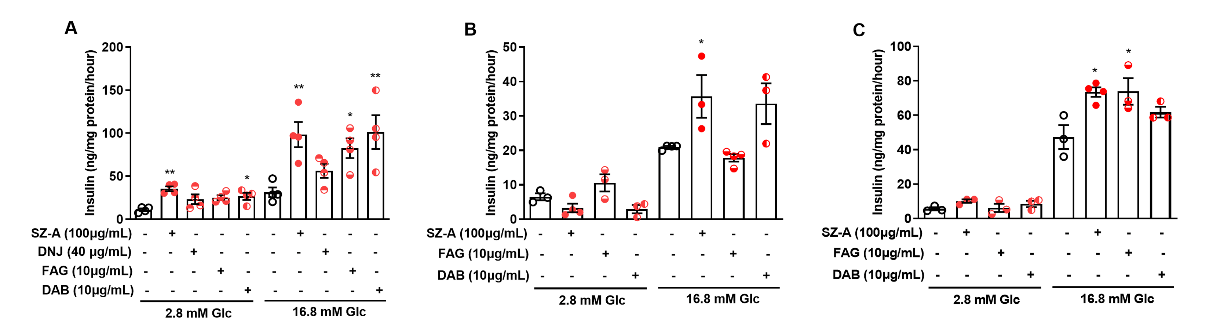


**Fig. S3.** The major effective fractions DAB and FAG promoted insulin secretion in MIN6 cells and mouse islets. Glucose-stimulated insulin secretion assay in MIN6 cells (A), islets of C57BL/6J mice (B) and islets of spontaneous type 2 diabetic KKA^y^ mice (C) at glucose concentrations of 2.8 mM and 16.8 mM. All data are expressed as the mean ± SEM (n=3-4 replicates per group). *P<0.05, **P<0.01, vs. the vehicle group.


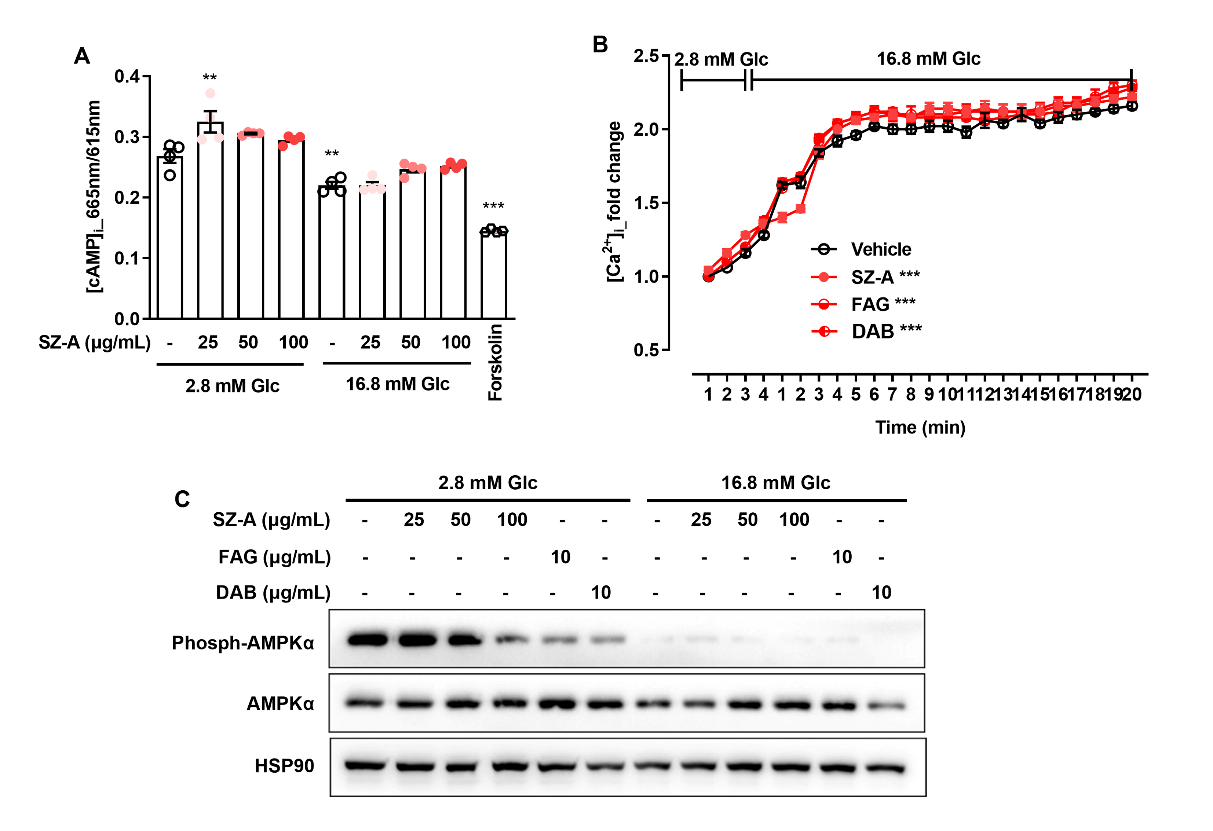


**Fig. S4.** SZ-A did not increase [cAMP]_i_, and the major effective fractions DAB and FAG enhanced glucose-dependent [Ca^2+^]_i_ changes and inhibited AMPKα phosphorylation in MIN6 cells. (A) The cytoplasmic concentration of cAMP in MIN6 cells treated with 25, 50, or 100 μg/mL SZ-A or vehicle at glucose concentrations of 2.8 mM and 16.8 mM. Forskolin is an adenylyl cyclase activator. All data are expressed as the mean ± SEM (n=4 replicates in each condition). **P<0.01, ***P<0.001, vs. the vehicle group at 2.8 mM glucose. (B) The changes in the [Ca^2+^]_i_ of MIN6 cells were labeled by Fluo4-AM when MIN6 cells were treated with 100 μg/mL SZ-A, 10 μg/mL FAG, 10 μg/mL DAB or vehicle while elevating the glucose concentration from 2.8 mM to 16.8 mM. All data are expressed as the mean ± SEM (n=5 replicates in each condition). ***P<0.001, vs. the vehicle group. (C) AMPKα expression level and AMPKα phosphorylation level in MIN6 cells incubated with 100 μg/mL SZ-A, 10 μg/mL FAG, 10 μg/mL DAB or vehicle at glucose concentrations of 2.8 mM and 16.8 mM for 1 h (n=3 replicates in each group).


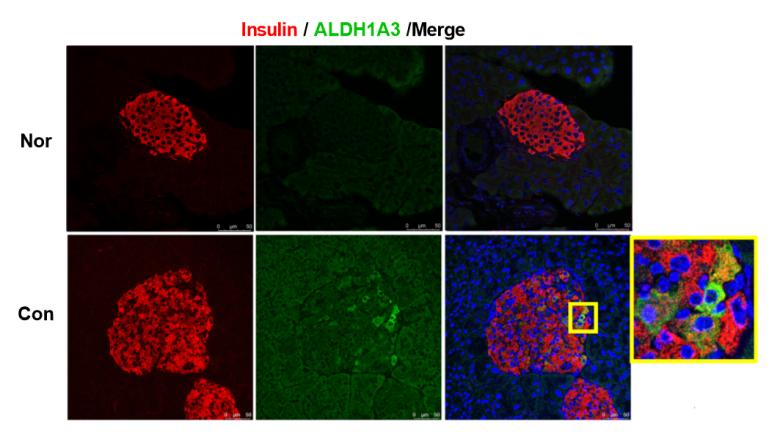


**Fig. S5.** Immunohistochemistry of the pancreas of normal C57BL/6J mice (Nor) and diabetic KKA^y^ mice (Con) (n=5 mice per group). β Cells were labeled with insulin antibody (red), dedifferentiated cells were labeled with ALDH1A3 antibody (green).


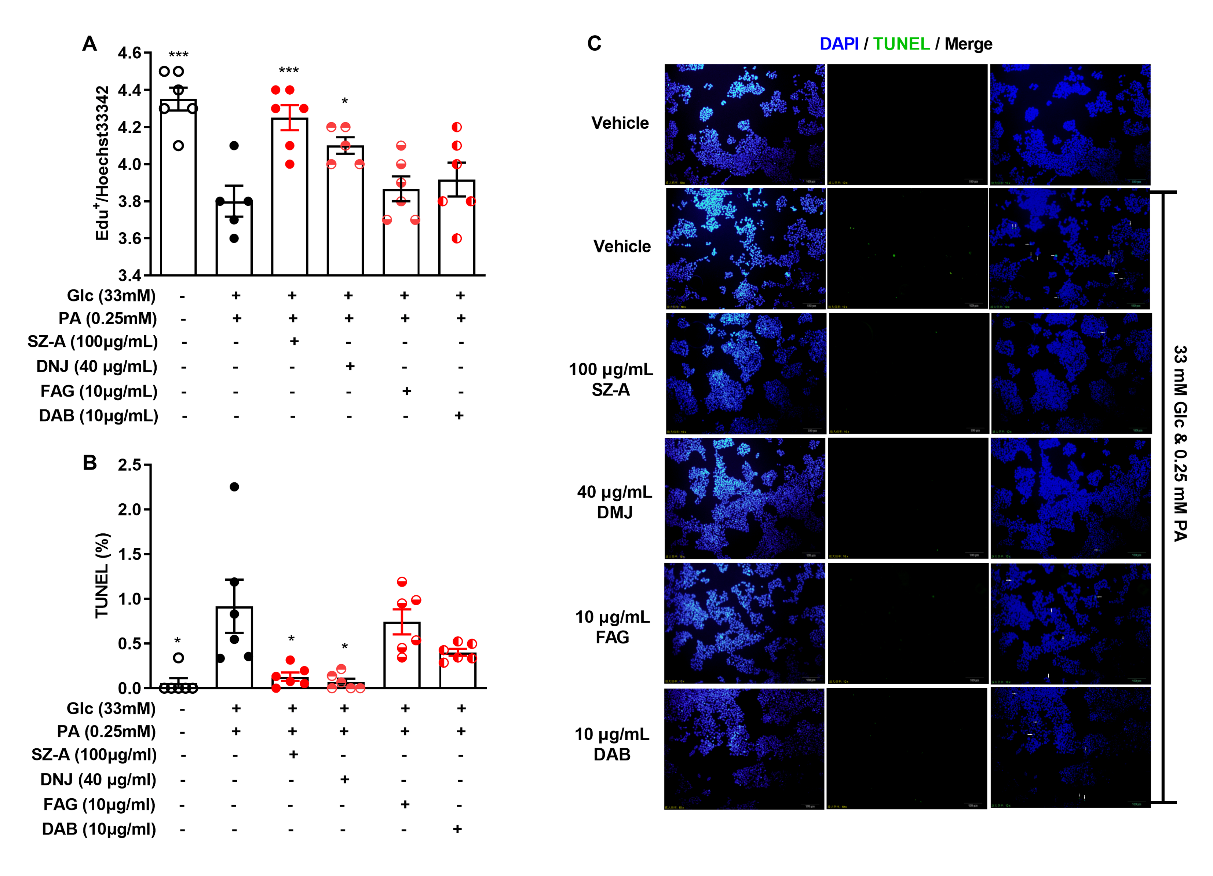


**Fig. S6.** The major effective fraction of DNJ attenuated apoptosis in high glucose- and PA-treated MIN6 cells. (A) MIN6 cells were treated with high glucose and PA and 100 μg/mL SZ-A, 40 μg/mL DNJ, 10 μg/mL FAG, 10 μg/mL DAB or vehicle for 24 h. The EdU assay was performed in MIN6 cells. (B-C) MIN6 cells were treated under the same conditions for 72 h. (B) The ratio of TUNEL-positive cells/cell nucleus. (C) Nuclei were labeled with DAPI (blue), and apoptotic bodies were marked by TUNEL (green). All data are expressed as the mean ± SEM (n=5-6 replicates per group). *P<0.05, ***P<0.001, vs. the Glc+PA group.


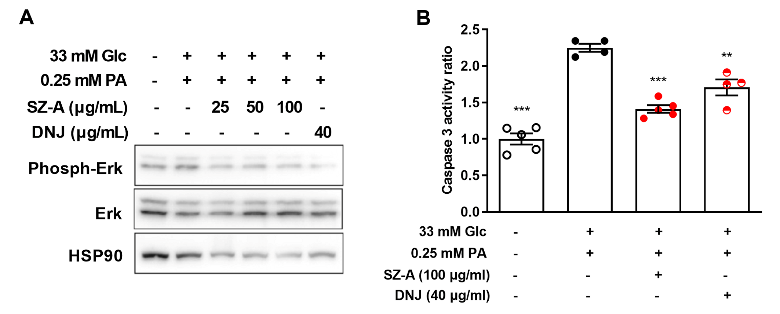


**Fig. S7.** The major effective fraction of DNJ decreased Erk1/2 phosphorylation and caspase 3 activity in high glucose- and PA-treated MIN6 cells. MIN6 cells were treated with high glucose and PA and 100 μg/mL SZ-A, 40 μg/mL DNJ or vehicle for 24 h. (A) Erk1/2 expression levels and Erk1/2 phosphorylation levels in MIN6 cells (n=3 replicates in each condition). (B) Caspase 3 activity ratio in MIN6 cells (n=4-5 replicates in each condition). **P<0.01, ***P<0.001, vs. the Glc+PA group.
